# Vital Signs During the COVID-19 Outbreak: A Retrospective Analysis of 19,960 Participants in Wuhan and Four Nearby Capital Cities in China

**DOI:** 10.5334/gh.913

**Published:** 2021-07-13

**Authors:** Jing-Wei Li, Yu-Tao Guo, Gian Luca Di Tanna, Bruce Neal, Yun-Dai Chen, Aletta E. Schutte

**Affiliations:** 1 Department of Cardiology, People’s Liberation Army General Hospital, Beijing, CN; 2 The George Institute for Global Health, Faculty of Medicine, University of New South Wales, NSW, Sydney, AU; 3 Department of Cardiology, Xinqiao Hospital, Army Military Medical University, Chongqing, CN; 4 Imperial College London, London, UK; 5 Medical School, People’s Liberation Army General Hospital, Beijing, CN

**Keywords:** lockdown, sleep, anxiety, depression, physical activity

## Abstract

**Background:**

The implications of city lockdown on vital signs during the COVID-19 outbreak are unknown.

**Objective:**

We longitudinally tracked vital signs using data from wearable sensors and determined associations with anxiety and depression.

**Methods:**

We selected all participants in the HUAWEI Heart Study from Wuhan and four nearby large provincial capital cities (Guangzhou, Chongqing, Hangzhou, Zhengzhou) and extracted all data from 26 December 2019 (one month before city lockdown) to 21 February 2020. Sleep duration and quality, daily steps, oxygen saturation and heart rate were collected on a daily basis. We compared the vital signs before and after the lockdown using segmented regression analysis of the interrupted time series. The depression and anxiety cases were defined as scores ≥8 on the Hospital Anxiety and Depression Scale depression and anxiety subscales [HADS-D and HADS-A] in 727 participants who finished the survey.

**Results:**

We included 19,960 participants (mean age 36 yrs, 90% men). Compared with pre-lockdown, resting heart rate dropped immediately by 1.1 bpm after city lockdown (95% confidence interval [CI]: –1.8, –0.4). Sleep duration increased by 0.5 hour (95% CI: 0.3, 0.8) but deep sleep ratio decreased by 0.9% (95% CI: –1.2, –0.6). Daily steps decreased by 3352 steps (95% CI: –4333, –2370). Anxiety and depression existed in 26% and 17% among 727 available participants, respectively, and associated with longer sleep duration (0.2 and 0.1 hour, both p < 0.001).

**Conclusions:**

Lockdown of Wuhan in China was associated with an adverse vital signs profile (reduced physical activity, heart rate, and sleep quality, but increased sleep duration). Wearable devices in combination with mobile-based apps may be useful to monitor both physical and mental health.

**Clinical trial registration:**

The trial is registered at Chinese Clinical Trial Registry (ChiCTR) website (ChiCTR-OOC-17014138).

## Introduction

Wuhan is the capital of the Hubei province in China, with more than 11 million residents. On 23 January 2020, the Chinese government initiated an unprecedented public health intervention to confine the epidemic of COVID-19 by shutting down all transportation in and out of Wuhan. Social distancing was practiced and outdoor activities were limited to the extreme [1]. To the 15^th^ of March 2020, there were 2,469 deaths in Wuhan, with 3,213 total deaths across the country, whereas cumulative confirmed cases were 67,798 in Hubei, and countrywide 80,860. With the majority of countries in the world applying strict lockdown and social distancing, public anxiety and depression may result from reported increases in death in regions that are severely affected. It remains largely unknown what the effects of these measures are on key indicators of health with no high quality data available from China [2,3]. With unique data available from smart wearable devices in populations residing in Wuhan and four regional cities during the COVID-19 outbreak, we were able to prospectively describe vital signs (heart rate, sleep, steps, oxygen saturation) across two months spanning the period before and after lockdown. We also established whether these measures are associated with questionnaire derived anxiety and depression status.

## Methods

The HUAWEI Heart Study was developed to track patients’ cardiovascular health using HUAWEI smart technology around China in collaboration with the Department of Cardiology at the People’s Liberation Army General Hospital. The study was initiated on 25 October 2018 and recruited 1,041,019 participants by 23 January 2020. Age, sex and body mass index (BMI) was collected by self-report through the software at the time of the participants’ inclusion. Inclusion criteria were use of a Huawei mobile phone (Android 5.0 or higher) and one of the following smart devices: Huawei Watch GT (Version 1.0.3.52 or higher), Honor Watch (Version 1.0.3.52 or higher) or Honor Band 4 (Version 1.0.0.86 or higher). Exclusion criteria included age <18 years, and inability to use a smart phone or the devices. The study was approved by the Central Medical Ethics Committee of the Chinese PLA General Hospital (Approval number: S2017-105-02) and registered at the Chinese Clinical Trial Registry (ChiCTR) website (ChiCTR-OOC-17014138). Participants were able to freely download the application from the HUAWEI App store. All volunteers who were interested in the study were informed of the study procedures and gave their informed consent before entering the study. Full data is available upon request and following approval by the steering committee.

Participants were included in the present study if they were part of the HUAWEI Heart Study and resident in Wuhan or one of four large nearby capital cities, namely Guangzhou, Chongqing, Hangzhou or Zhengzhou. Participants were also required to have data available for measurements taken between 26 December 2019 and 21 February 2020.

## Sleep, activity, stress and oxygen saturation

Sleep status was determined based on an acceleration sensor (ACC, actigraphy) and photoplethysmogram (PPG), which assessed six variables: sleep duration, time when fell asleep, wake up time and deep sleep time. Sleep duration, which is the difference between wake up time and time when falling asleep, and deep sleep ratio, which is the ratio of deep sleep time with sleep duration, were also assessed in the current analysis. We also extracted daily steps based on ACC and GPS signal. Oxygen saturation (SpO2) data were measured based on a blood oxygen saturation sensor, which could be activated by the participants. In a sleep apnea sub-study, which used the device to measure sleep in participants with potential sleep apnea, participants were encouraged to use this function (SpO2).

## Atrial fibrillation detection

An arrhythmia screening App was developed based on the Android Operating System (Google, Mountain View, California) for the HUAWEI Heart Study. Participants were required to install this software as part of their involvement in the study. Participants could initiate rhythm monitoring with the arrhythmia screening App using their smart device. Screening for irregular pulse waves was done with continuous or periodic measurements using the PPG algorithm. Individuals could initiate active measurements at rest and 45-second PPG recordings would then be collected. Periodic measurements were automatically taken every 10 minutes, with 60-second PPG signals collected. Atrial fibrillation (AF) was detected based on a combination of morphology and frequency analysis of the pulse waveform as previous described [4]. A machine learning method ‘boosting’ was used to train the model to screen AF prior to study commencement. Sensitive features extracted from the waveforms and the peak-to-peak intervals of the PPG were used in the model. The peak-to-peak intervals of PPG were uniform for sinus rhythm data but chaotic for AF episodes [5]. The sensitivity and specificity of the smart band PPG for detection of AF have previously been showed to be 95.36% (95% CI 92.00%–97.40%) and 99.70% (95% CI 98.08%–99.98%), respectively [6].

## Questionnaire on HADS anxiety and depression

On 21 February we invited all participants to complete the HADS questionnaires using the mobile app to determine the anxiety and depression status of all participants [7]. We received responses from a limited sample of 772/19,960 (3.87%) participants. We defined depression and anxiety asa HADS score of 8–21; and ‘normal mood’ as a HADS score of 0–7, as previously described [8].

## Statistical analysis

Continuous variables were presented as a means and standard deviations (SD) and count data as numbers (proportions). Daily averages of vital signs or proportions with AF were calculated using all data available on that day.

The pre-lockdown period was defined as from 26 December 2019 to 22 January 2020 (one month before city lockdown). The post-lockdown period spanned 23 January 2020 to 21 February 2020. Segmented least squares regression models were fitted to the daily series with parameters for intercept, pre-lockdown trend, and changes in level and trend after the lockdown, assuming linearity of the trend lines within each segment. We tested for up to six-order autocorrelation, using the Durbin-Watson statistic as a measure of autocorrelation [9]. Using the parameter estimates resulting from the model, we estimated the difference between observed and expected vital sign values and proportions of people at the end of the pre-lockdown period and after 30 day of lockdown. The mean difference between observed and expected proportions and their 95% CI were reported.

Next, the associations between anxiety/depression derived from HADS questionnaire and outcomes were analysed. For continuous vital sign outcomes with repeated measurements, a mixed model was used. Differences between anxiety/depression in the longitudinal changes from lockdown in each vital sign were analysed individually by using a mixed effects repeated-measures model to establish the effect of each category of anxiety/depression on the variable of interest. The model for each variable included as covariates the baseline age, sex, BMI, and date as fixed effects. For binary AF results, logistic regression was conducted, adjusted for age, sex and BMI. The participants were regarded as having an AF event if at least one episode of AF was detected by the wearable device from lockdown to 21 February 2020 [10].

A 2-sided P value <0.05 was considered statistically significant. Complete case analyses have been performed with no imputation applied. All statistical analyses were performed using SAS version 9.40 (SAS institute, Cary, NC) and Stata 16.0 (StataCorp, College Station, TX, USA).

## Results

**Figure 1:**
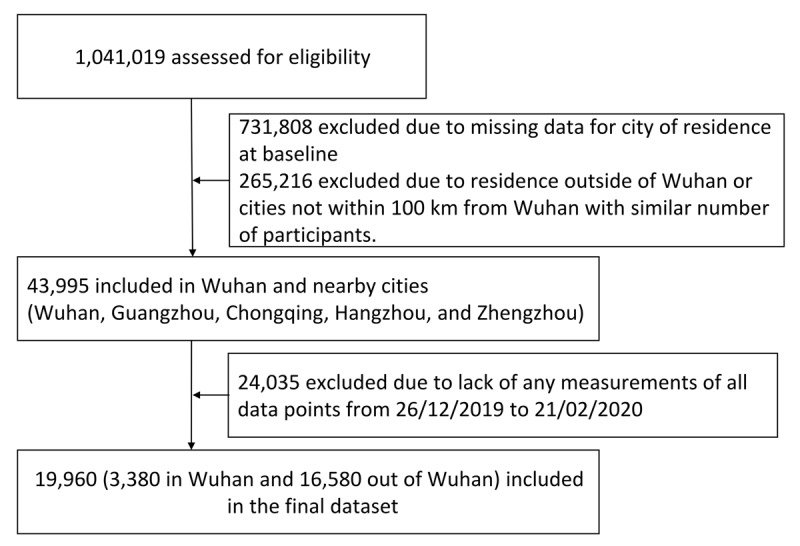
Participants flow chart.

Of the 19,960 participants included in this study, who have records from 26 December 2019 to 21 February 2020, 3,380 were from Wuhan and 16,580 from the nearby cities of Chongqing, Guangzhou, Hangzhou and Zhengzhou (Figure 1). Among them, 13,575 participants had 481,639 records of heart rate; 14,491 participants had 446,786 records of sleep; 15,862 participants had 793,078 records of steps; 3,809 participants had 61,661 records of oxygen saturation; and 15,740 participants had 187,063 records of AF. Participants in this study were relatively young (average age of 36 yrs) and the majority were men (90%). Baseline characteristics are balanced between Wuhan and other cities (Table 1).

**Table 1:** Characteristics before city lockdown of participants in and outside of Wuhan.

	All (n = 19,960)	Wuhan (n = 3,380)	Out of Wuhan (n = 16580)

Age, yrs	35.7 (11.3)/19960	35.7 (11.6)	35.7 (11.3)
Men, N (%)	17948/19960 (89.9%)	3056 (90.4%)	14892 (89.8%)
Body mass index, kg/m^2^	24.1 (3.7)/19733	24.2 (3.7)	24.1 (3.7)
Pre-lockdown data from device			
Atrial fibrillation, N (%)	40/11985 (0.3%)	6 (0.3%)	34 (0.3%)
Sleep duration, hours	7.1 (1.1)/12894	7.2 (1.1)	7.1 (1.1)
Deep sleep ratio, %	25.3 (7.8)/12894	25.6 (7.8)	25.3 (7.8)
Resting heart rate, bpm	66.9 (4.7)/12176	66.8 (4.5)	66.9 (4.7)
Steps, N	9542.1 (3703.9)/14601	9267.6 (3616.7)	9602.1 (3720.1)
Oxygen saturation, %	96.3 (0.5)/2463	96.3 (0.5)	96.3 (0.5)

Data are presented as mean (standard deviation) or No. (Proportions). BPM, beats per minute. The pre-lockdown atrial fibrillation, sleep time, heart rate, steps are mean levels from 26 December 2019 to city lockdown (22 January 2020).

## Vital signs and atrial fibrillation

**Figure 2:**
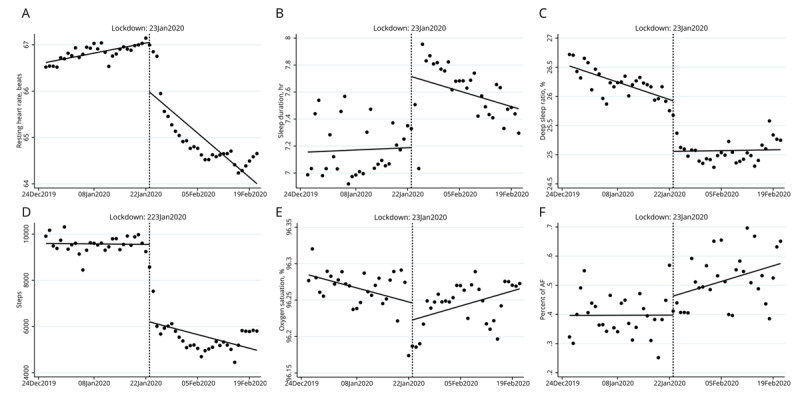
**Time series of vital signs before and after lockdown. Resting heart rate (a), Sleep duration (b), deep sleep ratio (c), steps (d), oxygen saturation (e) and proportion of atrial fibrillation (f)**. Fitted trend lines show predicted values from the segmented regression analysis for vital signs or AF before and after lockdown.

Resting heart rate decreased immediately by 1 bpm after city lockdown, with a slope decrease of 0.08 bpm per day compared to pre-lockdown. Sleep duration increased by 0.5 hour but was accompanied by a slight decreased in deep sleep ratio (–0.87%, 95% CI [–1.16, –0.58], p < 0.001). Daily steps also decreased substantially by 3352 steps (95% CI [–4333, –2370], p < 0.001). Oxygen saturation did not change (–0.02%, 95% CI [–0.06, 0.01], p = 0.159) and a slight increase in slope of 0.003% per day compared with pre-lockdown was observed. We found no change in the frequency of atrial fibrillation (Figure 2 and Table 2). Moreover, there was no difference in any of the other outcomes after lockdown between Wuhan and nearby cities (e-Table 1).

**Table 2:** Segmented regression analysis of change in vital signs and atrial fibrillation after the lockdown.

	Change in level				Change in slope			

	Mean	95% CI		P value	Mean	95% CI		P value

Resting heart rate, beats	–1.073	–1.757	–0.389	0.003	–0.084	–0.121	–0.046	<0.001
Sleep duration, hr	0.526	0.280	0.772	<0.001	–0.009	–0.023	0.005	0.183
Deep sleep ratio, %	–0.868	–1.157	–0.578	<0.001	0.022	0.006	0.038	0.008
Steps	–3352	–4333	–2370	<0.001	–40.72	–94.00	12.56	0.131
Oxygen saturation, %	–0.023	–0.056	0.009	0.159	0.003	0.001	0.005	0.010
Atrial fibrillation, %	0.065	–0.042	0.173	0.229	0.004	–0.002	0.010	0.207

CI, confidence interval.

## Anxiety and depression with vital signs

Overall, the HADS anxiety score was 5 [3,8] and the HADS depression score was 4 [2,7] in the subset of 727 participants who completed the HADS questionnaire. There was no difference in mean HADS scores between Wuhan and nearby cities (Wuhan vs. nearby cities, HADS anxiety score: 5 [4,7] versus 3 [2,7], p = 0.724; HADS depression score: 3 [2,7] versus 4 [2,7], p = 0.755). The response rate was 1% in Wuhan and 4% in other cities. The participants with possible anxiety or depression consisted of 26% (n = 185) and 17% (n = 126) of the total population, without any evidence of differences in prevalence between Wuhan and nearby capital cities (p = 0.400 for anxiety and p = 0.725 for depression, respectively). Anxiety (yes vs. no) was associated with longer sleep duration (0.2 hr longer) and a negligible (albeit statistically significant) increase in deep sleep ratio (0.008% increase). Depression also showed similar trends for sleep duration and deep sleep ratio and was also associated with less activity (–494 steps) and a negligible decrease in oxygen saturation (–0.09%) (Table 3).

**Table 3:** Segmented regression analysis for the difference of vital signs after lockdown according to anxiety and depression (Yes vs. No). The anxiety or depression were defined as >7 in HADs anxiety or depression score.

	Anxiety				Depression			

	Difference	95% CI		P value	Difference	95% CI		P value

Resting heart rate, beats	–0.036	–0.233	0.161	0.719	0.199	–0.028	0.426	0.086
Sleep duration, hr	0.207	0.146	0.269	<0.001	0.137	0.067	0.207	<0.001
Deep sleep ratio, %	0.008	0.004	0.011	<0.001	0.019	0.015	0.023	<0.001
Steps	–153.867	–317.014	9.279	0.065	–493.903	–681.783	–306.023	<0.001
Oxygen saturation, %	0.028	–0.009	0.066	0.141	–0.092	–0.137	–0.046	<0.001

CI, confidence interval.

## Discussion

Our study including 19,960 participants from Wuhan and four nearby cities found that the lockdown during the COVID-19 epidemic was associated with an acute increase in sleep quantity, but reduced sleep quality, resting heart rate and physical activity. Anxiety and depression were related to sleep quantity, steps, resting heart rate and oxygen saturation but were not different between the participants from Wuhan and the participants from nearby cities.

A study from Italy including 1,310 young participants has found lockdown caused by COVID-19 to be associated with self-reported longer sleep quantity and lower sleep quality [11]. Another study including about 100 participants in Wuhan reported that every 1,000 confirmed COVID-19 cases in Wuhan city reported by the media was associated with an estimated loss of 39 minutes of sleep [12], with anxiety likely to be part of the reason [13]. Changes in objective sleep quantity and sleep quality were verified in our study with a large number of participants, but our results do not support anxiety and depression as part of the reason, since the anxiety and depression were associated with longer sleep duration. The change in sleep patterns may simply reflect that people have more time to sleep due to the lockdown requiring them to spend much more time in their homes. We found that heart rate reduced steeply after lockdown, which is likely due to the associated large reduction in physical activity [14,15]. The lockdown greatly restricted general movement as reflected by the daily steps count. Previous studies reported that lockdown may decrease incidence of AF during the COVID-19 outbreak. A nationwide Danish registry including 5.6 million people found that lockdown led to a 47% drop in registered new-onset AF cases, and noted that the risk of undiagnosed AF patients developing complications may potentially translate into serious adverse health outcomes [16]. A Germany study in 66 Helios hospitals also found that emergency hospital admissions for AF decreased by about 20%, accompanied by a reduction in interventional treatments for AF during the Covid-19 pandemic [17]. We did not observe a significant increase or decrease in AF though the incidence of AF in our study was very low, likely due to the young age of the participants, who would also be unlikely to fulfil criteria for AF management defined by international guidelines.

Previous research reported that approximately 1 in 4 participants reported depression and about 1 in 6 reported anxiety during COVID-19 pandemic [18].

Wearable devices are ideal tools to actively track vital signs and arrhythmias. Using these devices to collect data on vital signs and arrhythmia such as AF among a large population enhanced our ability to monitor health during the lockdown, as lockdown did not permit usual face-to-face data collection [19]. Wearable device data were also found to improve real-time surveillance of influenza-like illness in the USA [20] and have been used to monitor patients exposed to the Ebola virus, saving time for health workers and reducing health workers exposure to pathogens [21]. Recently, biometric shirts capable of continuously measuring vital signs, including temperature, respiratory effort, and cardiac activity have been designed with the goal of better tracking the evolution of COVID-19 and its effects on lung function [22]. Moreover, contact tracing and support via smartphones has been widely used during the COVID-19 outbreak [23]. Mobile phone applications in combination with wearable devices and ‘chatbots’ could potentially assist patients with COVID-19 and those requiring other routine clinical services [24].

## Limitations

Our findings add to our understanding on how lives have been disrupted across the world for millions of people, and may provide insight to health ministries about interventions targeting physical and mental disturbance caused by COVID lockdowns [25]. Our study has several limitations. Firstly, the study population is greatly over-represented by young men due to their increased interest in using wearable devices. Secondly, there are many missing data because participants do not wear their devices or activate all functions at all times, though there is no strong reason to expect patterns of usage to be sustainedly different across geographies or over time. Thirdly, other variables could contribute to changes in the outcomes we measured in the context of a lockdown, including increased alcohol intake, inability to adhere to medication regimens and less control of other comorbidities like hypertension. Data to control for these factors were not available in this study. The HADS questionnaire was only conducted at one time point in a small proportion of our participants and the generalizability of the findings related to the HADS measures is therefore uncertain. In addition, we could not analyze the dynamic change of mental health during the lockdown process. Finally, we could not distinguish between those infected with COVID-19 and those that were uninfected in our study population, which prevents us from understanding how much of the changes in vital parameters are actually due to the ‘biological stress’ of infection vs the socio-psychological stress produced by the lockdown policy. Most participants outside of Wuhan are likely to not have been infected since more than 80% of confirmed COVID-19 cases were in Hubei province, and 70% in Wuhan, itself. Nonetheless only a small minority of Wuhan residents were infected and most study participants would not have been infected during the study period.

## Conclusion

In conclusion, we observed lockdown to be associated with reduced heart rate, physical activity, and sleep quality, but increased sleep duration. With increased popularity in the use of wearable devices, it may become an ideal population level surveillance tool when access to hospitals and laboratories are restricted.

## Additional Files

The additional files for this article can be found as follows:

10.5334/gh.913.s1 e-Figure 1. **Time series of vital signs before and after lockdown for Wuhan and nearby capital cities. Resting heart rate (a), Sleep duration (b), deep sleep ratio (c), steps (d), oxygen saturation (e) and proportion of atrial fibrillation (f).** Fitted trend lines show predicted values from the segmented regression analysis for vital signs or AF before and after lockdown in solid line (Wuhan) and dotted line (nearby capital cities).

10.5334/gh.913.s2 e-Table 1. Change in vital signs and atrial fibrillation after the lockdown between Wuhan and nearby capital cities.

10.5334/gh.913.s3 e-Table 2. Analysis of cumulative death (>1600 cases vs. ≤1600) with sleep duration, heart rate and atrial fibrillation according to different baseline characteristics using individual participant data.
