# Correction: Vital Signs During the COVID-19 Outbreak: A Retrospective Analysis of 19,960 Participants in Wuhan and Four Nearby Capital Cities in China

**DOI:** 10.5334/gh.1172

**Published:** 2022-12-20

**Authors:** Jing-Wei Li, Yu-Tao Guo, Gian Luca Di Tanna, Bruce Neal, Yun-Dai Chen, Aletta E. Schutte

**Affiliations:** 1 Department of Cardiology, People’s Liberation Army General Hospital, Beijing, CN; 2 The George Institute for Global Health, Faculty of Medicine, University of New South Wales, NSW, Sydney, AU; 3 Department of Cardiology, Xinqiao Hospital, Army Military Medical University, Chongqing, CN; 4 Medical School, People’s Liberation Army General Hospital, Beijing, CN

**Keywords:** COVID-19, Cardiology

## Abstract

This article details a correction to: Li J-W, Guo Y-T, Di Tanna GL, Neal B, Chen Y-D, Schutte AE. Vital Signs During the COVID-19 Outbreak: A Retrospective Analysis of 19,960 Participants in Wuhan and Four Nearby Capital Cities in China. Global Heart. 2021;16(1):47. DOI: http://doi.org/10.5334/gh.913.

## Correction

The original article^1^ was published without complete funding details. It listed one funder, the National Natural Science Foundation of China (H2501), National Key Research and Development Project of China (2018YFC2001200).

There was another funder missing from the original article, the Chinese Military Health Care (20BJZ26).

The originally listed funder covered expenses for enrolment and follow-up of patients, and the purchase and maintenance of necessary equipment. The second funder covered the costs of publication.

## Footnotes

http://doi.org/10.5334/gh.913.

