# Supplementary material for: Vital Signs During the COVID-19 Outbreak: A Retrospective Analysis of 19,960 Participants in Wuhan and Four Nearby Capital Cities in China
Source: Glob Heart. 2021 Jul 13;16(1):47. doi: 10.5334/gh.913 (PMC8284499; doi:10.5334/gh.913)

e-Figure 1. Time series of vital signs before and after lockdown for Wuhan and nearby capital cities. Resting heart rate (a), Sleep duration (b), deep sleep ratio (c), steps (d), oxygen saturation (e) and proportion of atrial fibrillation (f). Fitted trend lines show predicted values from the segmented regression analysis for vital signs or AF before and after lockdown in solid line (Wuhan) and dotted line (nearby capital cities).

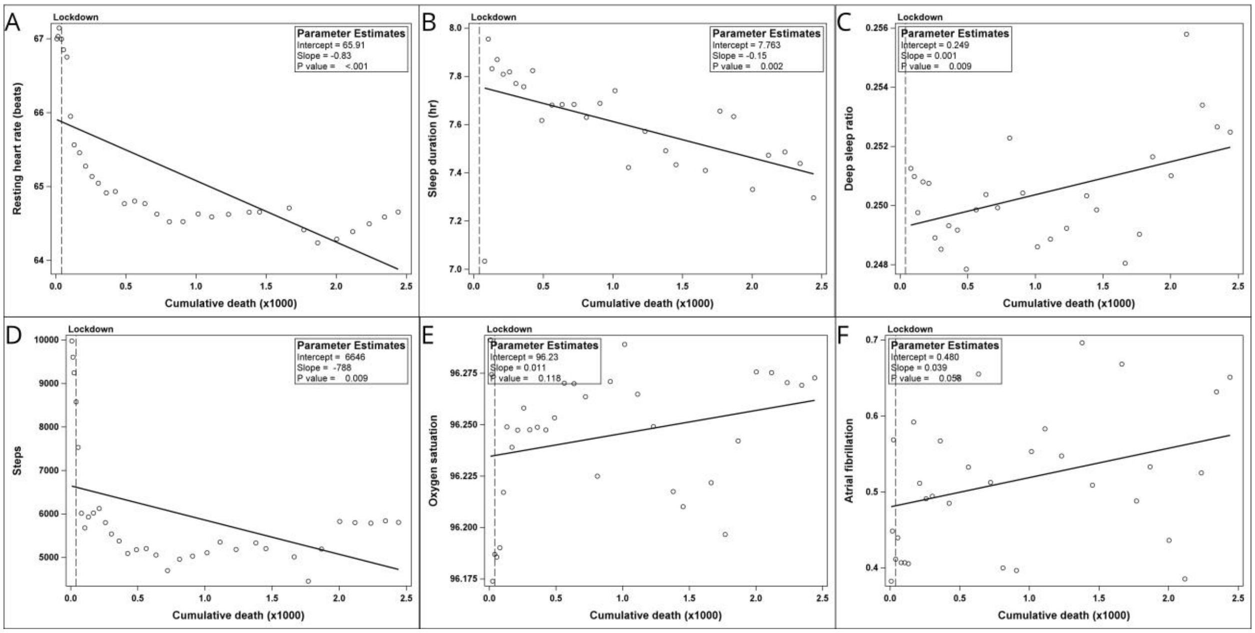

Supplement: e-Figure 1. — Time series of vital signs before and after lockdown for Wuhan and nearby capital cities. Resting heart rate (a), Sleep duration (b), deep sleep ratio (c), steps (d), oxygen saturation (e) and proportion of atrial fibrillation (f). Fitted trend lines show predicted values from the segmented regression analysis for vital signs or AF before and after lockdown in solid line (Wuhan) and dotted line (nearby capital cities). [file gh-16-1-913-s1.pdf]
