# Supplementary material for: Vital Signs During the COVID-19 Outbreak: A Retrospective Analysis of 19,960 Participants in Wuhan and Four Nearby Capital Cities in China
Source: Glob Heart. 2021 Jul 13;16(1):47. doi: 10.5334/gh.913 (PMC8284499; doi:10.5334/gh.913)
Supplement: e-Table 1. — Change in vital signs and atrial fibrillation after the lockdown between Wuhan and nearby capital cities. [file gh-16-1-913-s2.pdf]

e-Table 1. Change in vital signs and atrial fibrillation after the lockdown between Wuhan and nearby capital cities.

	Difference in level at lockdown				Difference in slope after lockdown			
	Mean	95% CI		P value	Mean	95% CI		P value
Resting heart rate, beats	-0.085	-1.025	0.855	0.858	-0.013	-0.063	0.037	0.604
Sleep duration, hr	-0.032	-0.464	0.400	0.883	0.003	-0.015	0.020	0.765
Deep sleep ratio, %	0.077	-0.367	0.522	0.731	0.004	-0.018	0.026	0.691
Steps	-712.4	-2103	678.0	0.312	15.72	-54.93	86.38	0.660
Oxygen saturation, %	-0.015	-0.064	0.035	0.564	0.001	-0.002	0.004	0.343
Atrial fibrillation, %	0.007	-0.277	0.291	0.962	0.005	-0.011	0.021	0.514

CI, confidence interval.
