# Supplementary material for: Vital Signs During the COVID-19 Outbreak: A Retrospective Analysis of 19,960 Participants in Wuhan and Four Nearby Capital Cities in China
Source: Glob Heart. 2021 Jul 13;16(1):47. doi: 10.5334/gh.913 (PMC8284499; doi:10.5334/gh.913)
Supplement: e-Table 2. — Analysis of cumulative death (>1600 cases vs. ≤1600) with sleep duration, heart rate and atrial fibrillation according to different baseline characteristics using individual participant data. [file gh-16-1-913-s3.pdf]

e-Table 2. Analysis of cumulative death (>1600 cases vs. ≤1600) with sleep duration, heart rate and atrial fibrillation according to different baseline characteristics using individual participant data.

subgroup	Sleep duration (hr)				Heart rate (bpm)				Atrial fibrillation*			
	**Coeff**	**95% CI**		**P**	**Coeff**	**95% CI**		**P**	**OR**	**95% CI**		**P**
All	**-0.546**	**-0.601**	**-0.490**	**<0.001**	**-2.285**	**-2.440**	**-2.131**	**<0.001**	1.66	0.77	3.61	0.200
**Age, yr**												
<40	-0.584	-0.652	-0.516	**0.001**	-2.104	-2.290	-1.918	0.114	2.78	0.29	26.55	0.475
≥40	-0.473	-0.567	-0.379		-2.605	-2.879	-2.331		1.54	0.71	3.34	
**Sex**												
Female	-0.484	-0.675	-0.293	0.853	-1.219	-1.755	-0.682	**<0.001**	1.91	0.42	8.82	0.280
Male	-0.552	-0.609	-0.494		-2.380	-2.541	-2.218		1.59	0.67	3.75	
**BMI**												
<25	-0.588	-0.656	-0.519	0.113	-2.094	-2.288	-1.899	**<0.001**	1.84	0.65	5.15	0.630
≥25	-0.474	-0.567	-0.381		-2.592	-2.845	-2.338		1.46	0.43	4.92	
**AF at baseline**												
No	-0.551	-0.617	-0.484	0.753	-2.408	-2.595	-2.222	0.590	7.18	4.01	12.86	**0.002**
Yes	-0.911	-1.790	-0.032		-3.072	-5.941	-0.203		1.77	0.43	7.35	

**Sleep duration, h**												
<7	-0.548	-0.634	-0.463	0.785	-2.340	-2.594	-2.086	0.323	1.90	0.34	10.52	0.811
≥7	-0.530	-0.602	-0.457		-2.223	-2.439	-2.007		2.32	0.81	6.63	
**Deep sleep ratio (%)**												
<25	-0.594	-0.700	-0.488	0.878	-3.558	-3.839	-3.276	**<0.001**	2.29	0.89	5.88	0.023
≥25	-0.518	-0.586	-0.450		-1.547	-1.747	-1.347		2.28	0.25	21.19	
**Resting heart rate, b.p.m**												
<65	-0.529	-0.626	-0.431	0.347	-0.129	-0.364	0.106	**<0.001**	2.16	0.41	11.56	0.995
≥65	-0.560	-0.638	-0.482		-3.239	-3.419	-3.059		2.17	0.77	6.12	
**Steps**												
<10000	-0.572	-0.650	-0.494	0.251	-2.350	-2.559	-2.140	**0.012**	2.15	0.40	11.54	0.820
≥10000	-0.504	-0.589	-0.419		-2.115	-2.361	-1.869		1.92	0.65	5.63	
**Oxygen saturation**												
<96	-0.411	-0.655	-0.168	0.866	-5.496	-6.161	-4.832	0.089	1.42	0.30	6.66	0.570
≥96	-0.666	-0.816	-0.517		-4.503	-4.900	-4.106		1.71	0.16	18.89	

. Adjusted for age, sex and BMI.

* Adjust for total measurement time from the device each day.
